# Neuromelanin‐MRI to Quantify and Track Nigral Depigmentation in Parkinson's Disease: A Multicenter Longitudinal Study Using Template‐Based Standardized Analysis

**DOI:** 10.1002/mds.28934

**Published:** 2022-02-15

**Authors:** Yue Xing, Abdul Halim Sapuan, Antonio Martín‐Bastida, Saadnah Naidu, Christopher Tench, Jonathan Evans, Gillian Sare, Stefan T. Schwarz, Sarah Al‐bachari, Laura M. Parkes, Sofia Kanavou, Jason Raw, Monty Silverdale, Nin Bajaj, Nicola Pavese, David Burn, Paola Piccini, Donald G. Grosset, Dorothee P. Auer

**Affiliations:** ^1^ School of Medicine Mental Health & Clinical Neurosciences Nottingham United Kingdom; ^2^ Sir Peter Mansfield Imaging Centre University of Nottingham Nottingham United Kingdom; ^3^ National Institute for Health Research, Nottingham Biomedical Research Centre Nottingham United Kingdom; ^4^ Division of Neurology Imperial College London London United Kingdom; ^5^ Department of Neurology and Neurosciences Clínica Universidad de Navarra Pamplona‐Madrid Spain; ^6^ Neurology Nottingham University Hospital Trust Nottingham United Kingdom; ^7^ Department of Radiology Cardiff and Vale University Health Board Cardiff United Kingdom; ^8^ Division of Neuroscience and Experimental Psychology, Faculty of Biology, Medicine and Health The University of Manchester, Manchester Academic Health Science Centre Manchester United Kingdom; ^9^ Lancaster Medical School, Lancaster University Lancaster United Kingdom; ^10^ Department of Neurology Lancashire Teaching Hospitals NHS Foundation Trust Preston United Kingdom; ^11^ Division of Neuroscience & Experimental Psychology School of Biological Sciences, Faculty of Biology, Medicine and Health, The University of Manchester, Manchester Academic Health Science Centre Manchester United Kingdom; ^12^ Population Health Sciences Bristol Medical School, University of Bristol Bristol United Kingdom; ^13^ Pennine Acute Hospitals NHS Trust Oldham United Kingdom; ^14^ Division of Neurology Salford Royal NHS Foundation Trust, Manchester Academic Health Science Centre, University of Manchester Manchester United Kingdom; ^15^ Spire Nottingham Hospital Nottingham United Kingdom; ^16^ Newcastle Magnetic Resonance Centre & Positron Emission Tomography Centre and Clinical Ageing Research Unit Newcastle University Newcastle upon Tyne United Kingdom; ^17^ Faculty of Medical Sciences The Medical School, Framlington Place, Newcastle University Newcastle upon Tyne United Kingdom; ^18^ Department of Brain Science Imperial College London London United Kingdom; ^19^ Institute for Neurological Sciences Queen Elizabeth University Hospital Glasgow United Kingdom

**Keywords:** neuromelanin; magnetic resonance imaging; longitudinal study; depigmentation; substantia nigra

## Abstract

**Background:**

Clinical diagnosis and monitoring of Parkinson's disease (PD) remain challenging because of the lack of an established biomarker. Neuromelanin‐magnetic resonance imaging (NM‐MRI) is an emerging biomarker of nigral depigmentation indexing the loss of melanized neurons but has unknown prospective diagnostic and tracking performance in multicenter settings.

**Objectives:**

The aim was to investigate the diagnostic accuracy of NM‐MRI in early PD in a multiprotocol setting and to determine and compare serial NM‐MRI changes in PD and controls.

**Methods:**

In this longitudinal case–control 3 T MRI study, 148 patients and 97 controls were included from six UK clinical centers, of whom 140 underwent a second scan after 1.5 to 3 years. An automated template‐based analysis was applied for subregional substantia nigra NM‐MRI contrast and volume assessment. A point estimate of the period of prediagnostic depigmentation was computed.

**Results:**

All NM metrics performed well to discriminate patients from controls, with receiver operating characteristic showing 85% accuracy for ventral NM contrast and 83% for volume. Generalizability using a priori volume cutoff was good (79% accuracy). Serial MRI demonstrated accelerated NM loss in patients compared to controls. Ventral NM contrast loss was point estimated to start 5 to 6 years before clinical diagnosis. Ventral nigral depigmentation was greater in the most affected side, more severe cases, and nigral NM volume change correlated with change in motor severity.

**Conclusions:**

We demonstrate that NM‐MRI provides clinically useful diagnostic information in early PD across protocols, platforms, and sites. It provides methods and estimated depigmentation rates that highlight the potential to detect preclinical PD and track progression for biomarker‐enabled clinical trials. © 2022 The Authors. *Movement Disorders* published by Wiley Periodicals LLC on behalf of International Parkinson and Movement Disorder Society

**Abbreviations**
BL: baseline
DaTscan: dopamine transporter imaging
dnNMc: dorsal nigral NM contrast
dnNMv: dorsal nigral NM volume
FU: follow‐up
NM: neuromelanin
nNMc: nigral NM contrast
nNMv: nigral NM volume
SNpc: substantia nigra pars compacta
tnNMv: template‐based nigral NM volume
vnNMc: ventral nigral NM contrast
vnNMv: ventral nigral NM volume
wnNMv: whole nigral NM volume

Parkinson's disease (PD) is the second most common neurodegenerative disorder, with progressive and disabling motor and nonmotor symptoms, but has no effective disease‐modifying treatment.^1^, ^2^ The lack of an established in vivo biomarker of PD brain pathology continues to delay clinical diagnosis to later stages of the disease with already‐advanced brain disease.^3^, ^4^ Dopamine transporter imaging with [123I]FP‐CIT (DaTscan) detects striatal dopaminergic deficit that improves diagnostic accuracy in clinically uncertain cases and possibly prodromal cases.^5^ Longitudinal studies demonstrated progressive striatal dopamine loss^6^ with a negative exponential or linear pattern of decline, which may be related to early loss of compensatory mechanisms,^7^ and phenotypic variation^8^ use of serial DaTscans is further limited by exposure to iodinizing radiation, cost, and availability.

Neuromelanin (NM)‐sensitive magnetic resonance imaging (MRI) is a novel technique that might address the unmet need for a reliable, early imaging marker of PD, as well as to track disease progression. NM is a pigmented by‐product of catecholamine synthesis, which accumulates over time in the substantia nigra pars compacta (SNpc) of healthy brains. In PD, the progressive loss of melanized neurons leads to SNpc depigmentation, a pathological hallmark of PD. NM‐sensitive MRI is therefore highly attractive as a biomarker of nigral degeneration in PD^9^, ^10^, ^11^ and the diagnostic value of NM‐MRI was established in several case–control studies.^11^, ^12^, ^13^, ^14^, ^15^, ^16^, ^17^, ^18^, ^19^, ^20^ However, there are important limitations of reported NM‐MRI findings to date due to single‐center settings, lack of consensus and standardization of analysis protocols, and unknown generalizability in a multiscanning‐platform and multiprotocol setting.^21^ Also, demographic factors modifying nigral NM in controls^22^ are under‐researched in PD. There is strong histopathological evidence for ventral predominant loss of melanized neurons,^3^, ^4^, ^23^, ^24^ but uncertainty regarding spatial specificity and the trajectory of brainstem depigmentation remains.^16^, ^20^, ^21^

Importantly, there are few longitudinal studies in PD. Two longitudinal NM‐MRI studies^25^, ^26^ did not include control arms and did not report serial assessments of NM change at the individual level. A very recent serial analysis of the study cohorts reported in Gaurav et al^25^ demonstrated significant NM volume loss in early and progressive PD, with no change in healthy control groups, but NM contrast increases in early PD and a trend decreases in progressive PD.^26^ The study revealed significant scanner effects and was further limited by manual definition of SN that has limited accuracy, requires much training and is time consuming, and lacked subregional analysis, which may explain the discrepant contrast results.^26^ Overall, NM‐MRI shows great promise as a diagnostic and serial progression marker for clinical trials in PD, yet lacks multisite evidence and standardized analysis tools.

Here, we present a large prospective multicenter case–control NM‐MRI study based on a standardized template‐based analysis aiming to (1) investigate the diagnostic accuracy of NM‐sensitive MRI in early PD in a multiprotocol setting and (2) determine and compare annualized serial NM‐MRI changes in patients and controls. Secondary study aims included the characterization of spatial dependencies and clinicodemographic associations of NM loss and estimation of the premotor depigmentation period.

## Patients and Methods

### Recruitment Source

The Parkinson's Magnetic Imaging Repository (PaMIR) study is a multicenter longitudinal case–cohort multimodal MRI study at 3 T, undertaken in tertiary care settings in the United Kingdom with co‐recruitment of patients from the Tracking Parkinson's study (www.trackingparkinsons.org.uk), a multicenter prospective study of patients diagnosed with PD (Supplementary Material 1.1). Additional patients were recruited from movement disorder clinics related to the research sites, and controls were recruited via multiple pathways (Supplementary Material 1.2). The study and subsequent amendments were approved by the Research Ethics Committee (REC), NHS REC 14/EM/0061, and local Research and Innovation (R&I) departments. All participants provided written informed consent and were selected according to the inclusion and exclusion criteria of the Tracking Parkinson's study in addition to the PaMIR criteria related to MRI scanning (Supplementary Material 1.3).

### Participants

**FIG 1:**
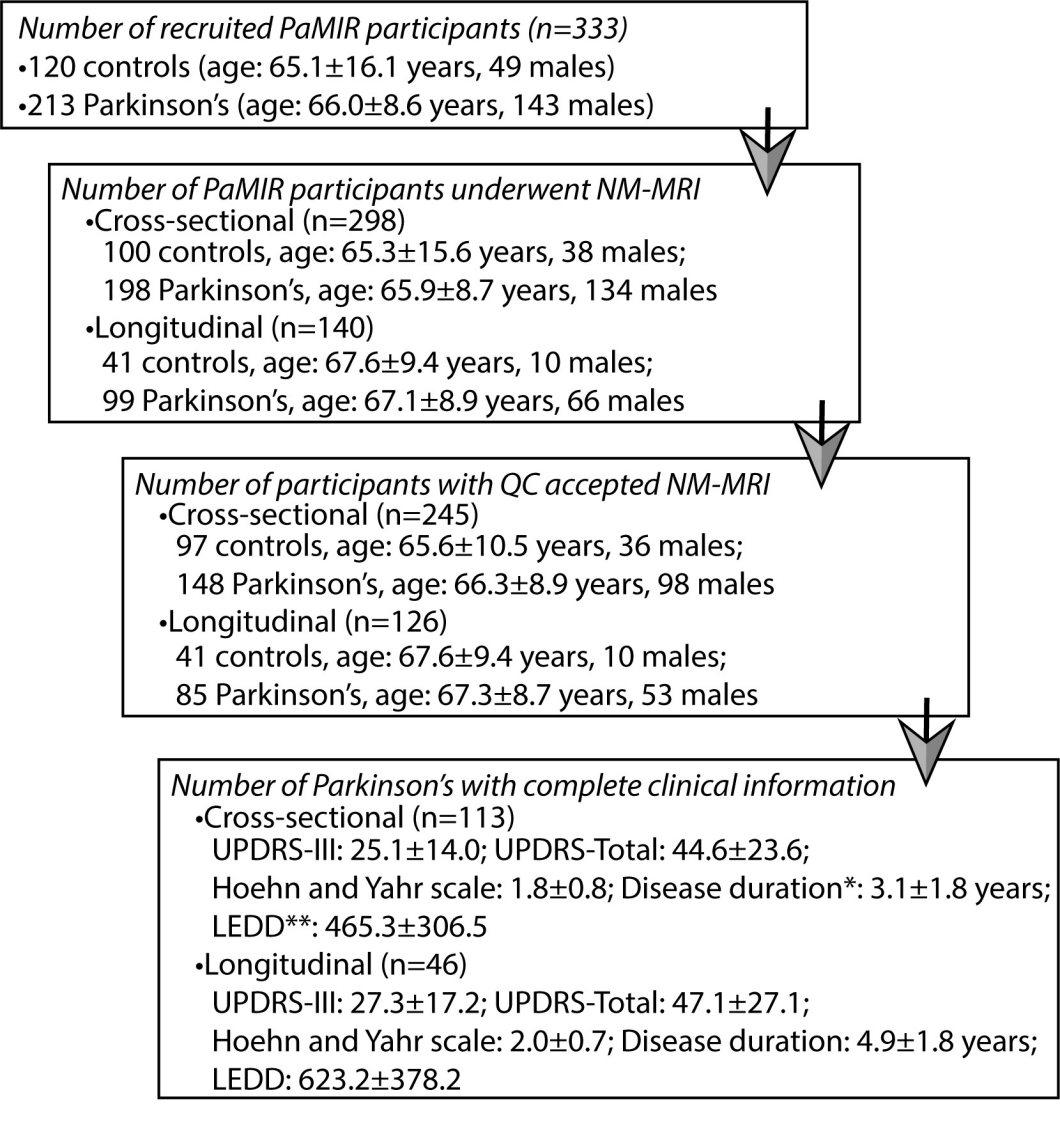
Flowchart of study progression at different stages and the demographic information of patients for baseline and follow‐up visits. All the values are presented as mean ± standard deviation (SD). *Disease duration was defined as the duration between the baseline scanning time and the time of diagnosis. **LEDD: levodopa equivalent daily dose. All the values are presented as mean ± SD.

Three hundred thirty‐three consenting participants were referred to five scanning centers from November 2014 to June 2019. A subgroup of participants were recalled and rescanned after 1.5 to 3 years. Numbers and demographic information are shown in Figure 1.

### Imaging Acquisition

NM‐MRI scans were acquired as part of the multimodal PaMIR protocol (Supplementary Material 1.4), with five protocols optimized for each scanner (Table S1; Figure S1).

### Collection of Clinical Information

Clinical information was recorded by either clinical research fellows or research nurses during MRI scanning, or, for Tracking Parkinson's participants, from their data repository. For correlation analysis of NM‐clinical findings, patients with missing data or whose clinical information exceeded 1 year from the scan date were excluded.

### Data Processing

#### Volumetry of NM‐Rich SN

To obtain comparable metrics of the size of NM‐rich SNpc across scanner protocols, and prospectively assess the generalizability of diagnostic accuracy based on a predefined threshold of depigmentation, we followed the previously reported normalization procedure.^18^ Briefly, the individual signal intensity thresholds were determined based on manual region of interest (ROI) analysis and applied a scanner and protocol‐specific adjustment factor to correct for protocol‐dependent sensitivity variation. The adjustment factor was derived from normative local control data sets to match the postmortem size of “normal” pigmented substantia nigra volume (~127 mm^3^).^27^ Finally, individual suprathreshold voxel counts were obtained for all participants and expressed in cubic millimeters (voxel size = 1 mm isotropic) for the whole SN (whole nigral NM volume) and separately for the “dorsal” and “ventral” parts of SN (equivalent to anterior and posterior in Schwarz et al^18^). We chose anatomical terminology over the previously used radiological terminology^18^ (“anterior” nNMv [nigral NM volume] equivalent to dorsal and “posterior” nNMv equivalent to ventral SN) for easier reference to the neuropathological literature^28^ and consistency with the ROI selection for contrast metrics. Reviewers were blind to the clinical status of the participants. Half of the data were randomly selected and analyzed again by the same operator (Y.X. with 8 years’ experience in brain‐imaging research) and a second reviewer (S.A. 3 years' experience) to obtain the intra‐rater (3‐month interval) and inter‐rater reliability of volume measurements. Intraclass‐ and interclass‐correlation analyses were applied to evaluate the repeatability of manual volumetry.

#### Template‐Based NM SN Contrast Assessment

**FIG 2:**
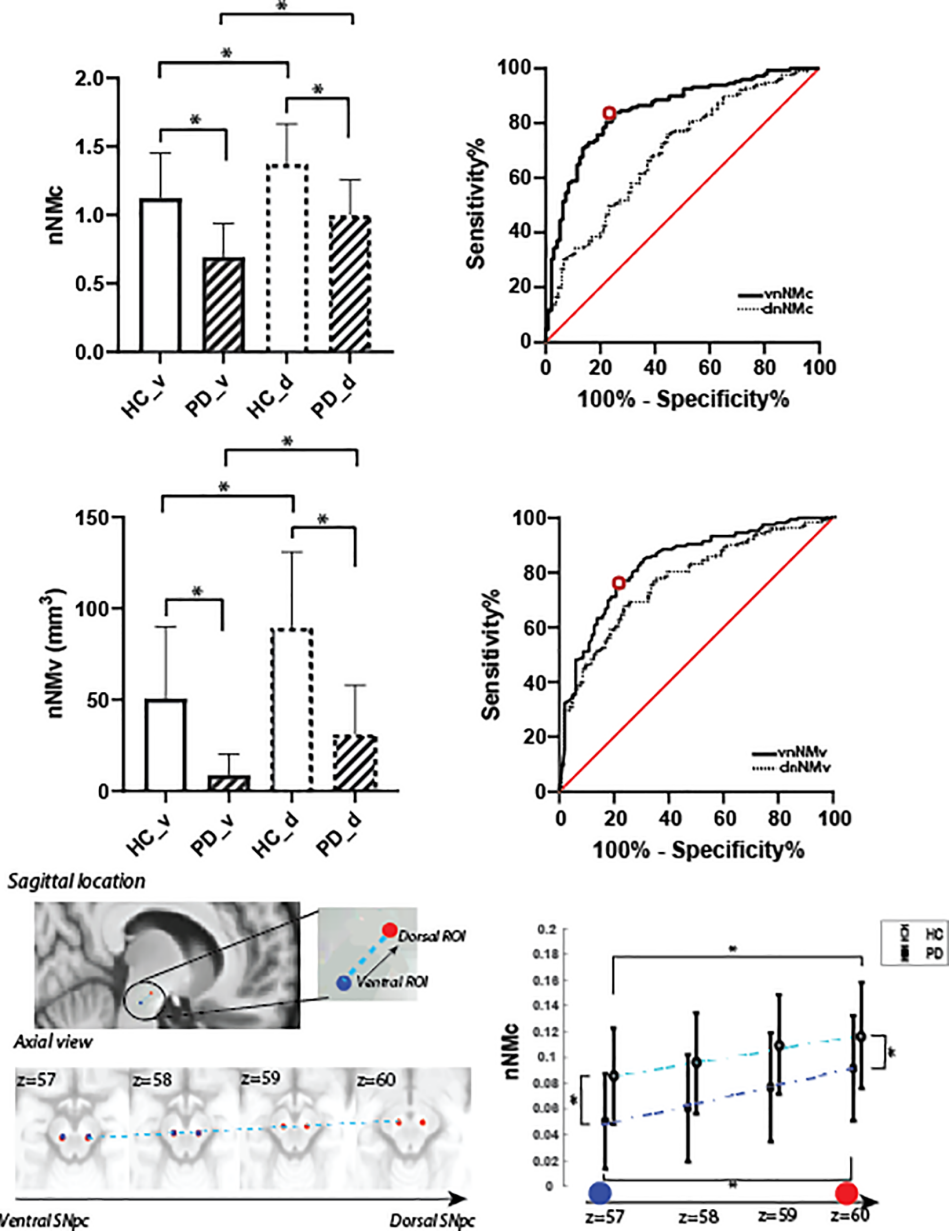
Diagnostic value of neuromelanin (NM) metrics in Parkinson's disease. Upper panel: normalized contrast background ratio; ventral (_v solid) and dorsal (d dashed) NM metrics show significant reduction in people with Parkinson's disease (PD, striped bars) versus controls (HC, white bars), with ROC (receiver operating characteristic) curves on the right. For unadjusted nNMc (nigral NM contrast), see Figure S5. Middle panel: normalized volumes and ROC curves. The red open circle marks the performance of generalization using the cutoff value from an independent data set. Bottom panel: group nNMcs (right) as a function of spatial location, shifting from the ventral to the dorsal part of SN (from blue to red ROIs on the left sagittal‐view image, whose locations are also illustrated in the axial images). The error bars represent the mean ± standard deviation (SD); * indicates that the difference between groups is statistically significant, adjusted for age and sex.

The signal of the brainstem outside SNpc was determined using an automated approach based on Bayesian classification (Supplementary Material 1.5; Figure S2). To calculate the ventral nigral NM contrast (vnNMc), a spherical ROI (kernel = 3‐mm‐diameter sphere) was placed in the ventral SNpc of the NM‐rich template following Gibb and Lees^23^ (see Fig. 2, and refer Supplementary Material 1.5 and Supplementary Figure 3 for coordinates and more details), and its mean signal (S_vSN_) was normalized to the mean signal of brainstem (S_BS_) according to vnNMc = (S_vSN_ – S_BS_)/S_BS_. The same approach was followed for the dorsal nigral NM contrast (dnNMc) using coordinates shown in Supplementary Material 1.5S and Figure S3. To then adjust for contrast differences resulting from technical variations between scanners and protocols, we normalized vnNMc using a local calibration factor by setting the mean dnNMc in controls to 1. For more details of the protocol and site effects and their normalization, see Supplementary Material 1.5 and Figure S4.

To characterize the spatial gradient of NMc in patients compared to controls, nigral NM contrast (nNMc) values were also obtained along the line that connects the centroids of dorsal and ventral ROIs using Bresenham's line algorithm (Fig. 2, bottom panel left).

#### Template‐Based NM Volumetry

To address the potential limitations of the manual steps in NM volumetry, we further modified the volumetric approach to derive fully automated template‐based NM volumetry as follows: using the template method described earlier, the background signal was determined as the mean intensity of voxels, with more than 50% posterior probability of being in the background area in each individual. The total template‐based normalized NM‐rich SN volume (tnNMv [template‐based nigral NM volume]) was obtained as voxel count in the region, with 30% posterior probability of being SN for all controls and exceeding individual intensity thresholds (manifolds of the standard deviations of the background voxel intensity as described earlier).

#### Longitudinal Measurement of NM‐MRI

Serial subnigral NM volume change was not analyzed due to a flooring effect as several patients showed near‐zero ventral nigral NM volume (vnNMv) at baseline (BL). For measuring the NM change between BL and follow‐up (FU) scans, we computed the individual annualized change rates for vnNMc, dnNMc, and tnNMv. We also estimated at group level the mean annualized percentage of reduction in vnNMc and dnNMc using a linear 100× (BL_nNMc‐FU_nNMc)/BL_nNMc/interval (years between the two scans) and estimated the onset of preclinical depigmentation in the vnNMc as the earliest‐affected nigral subregion.

### Statistical Analysis

Group‐specific effects of age and sex differences were reported. Multiple linear regression analysis was used for BL NM‐metric, adjusted for sex and age.

Diagnostic accuracy: effect sizes were calculated for both vnNMv and vnNMc as primary outcomes with dorsal contrast/volume and whole NM effects reported for completeness. Receiver operating characteristic (ROC) analysis was performed to determine the best cutoff values for controls versus disease based on the Youden index. Area under the curve, sensitivity, specificity, and negative and positive predictive values are reported. To assess generalizability, we applied the previously reported cutoff value of the vnNMv (referred to as posterior NM‐rich SNpc volume in^18^) to determine the independently out‐of‐sample cross‐validated sensitivity and specificity.

Serial changes: general linear mixed‐effects models with dependent variables, vnNMc, fixed effects age, sex, and FU time, and random subject factor on the intercept because of repeated measures were used to estimate the rate of change in the ventral SN. This was used to obtain a point estimate of premotor (disease onset) depigmentation period by extrapolation to the time where the patient's mean intersected the control mean NM contrast, adjusted for BL sex differences and age.

Clinical associations: to investigate the relationship between NM‐MRI metrics and clinical findings in PD, partial correlation analyses were performed, controlling for sex and age.

To account for the sex imbalance in the study cohort, the main tests were repeated in an age‐ and sex‐matched subsample.

Inference: to correct for multiple comparisons, *P*‐values of all the primary outcomes (diagnostic accuracy and serial change) were adjusted using the Benjamini–Hochberg method at a false discovery rate of 0.05. Where data were not normally distributed, we applied nonparametric tests. Significance was defined at α = 0.05. Statistical analysis was conducted using IBM‐SPSS for Windows and Matlab (more details are provided in Supplementary Material 1.6). The results are reported as either mean ± standard deviation (SD) or difference between mean and standard error.

## Results

Figure 1 shows the number of participants included at each stage of the study and the demographics for the patients included in BL and FU analyses. Quality control criteria and examples of excluded cases are provided in Supplementary Material 1.7 and Figure S5.

Reproducibility of semiautomated vnNMv was high, with intraclass coefficient (intra‐rater) of 0.86 (CI [confidence interval]: 0.80–0.92) and interclass coefficient (inter‐rater) of 0.84 (CI: 0.75–0.92).

### The Effect of Age, Sex, and PD on Nigral NM Metrics

Controls showed a significant age effect on ventral but not dorsal NM‐MRI metrics cross‐sectionally (Figure S6, upper panel). No age effect was observed on either metrics in the patient group.

Female sex was associated with larger vnNMv in controls (mean ± SD: women 33.5 ± 24.0 vs. men 20.2 ± 17.7 mm^3^, *P* < 0.01) and in patients (women 10.3 ± 11.3 vs. men 7.4 ± 10.5 mm^3^, adjusted *P* = 0.036). Similar sex effects were observed for dorsal nigral NM volume (dnNMv) (Figure S6, middle panel). Consistently, higher vnNMc was found in female controls (mean sex difference: 0.13 ± 0.21, adjusted *P* = 0.015) and female patients (mean difference 0.13 ± 0.003, adjusted *P* < 0.00001), but no sex difference was identified in dnNMc (Figure S6, bottom panel).

### Diagnostic Accuracy of NM Metrics in PD

Normalized vnNMv and vnNMc were lower in patients compared to controls (adjusted *P* < 0.0003), controlling for sex and age (volume mean difference: 20.4 mm^3^, 95% CI: 16.3–24.4 mm^3^, and contrast mean difference: 0.025 and 95% CI: 0.019–0.30) (Fig. 2 upper and middle panel). The effect size was nominally higher for vnNMc with Hedges' g^29^ of 1.44 (95% CI: 1.15–1.73) compared to vnNMv 1.24 (95% CI: 1.00–1.51).

Post hoc tests in a sex‐matched subsample confirmed significant between‐group differences for both vnNMc and vnNMv (see Figure S7 and Table S2 for details).

ROC curves (Fig. 2) using site‐specific normalized vnNMc and vnNMv data exhibited very good diagnostic accuracy (Table 1) for both metrics with nominally better performance of contrast ratios. The accuracy when generalizing previously published cutoffs of vnNMv (10.72 mm^3^) from our independent multiscanner data set^18^ to the current multicenter data set remained high (Table 1). Figure 2 shows the spatial profile of nNMc along the main SNpc axis, demonstrating an increasing ventral–dorsal contrast in both groups, with an apparent steeper slope in patients (slope = 0.014 in patients vs. 0.01 in controls) and consistently lower nNMc across the SNpc.

**TABLE 1:** Discrimination accuracy of NM metrics in the SNpc

	Accuracy (95% CI)	Sensitivity (95% CI)	Specificity (% 95% CI)	Positive predictive value (% 95% CI)	Negative predictive value (% 95% CI)
vnNMc	0.85 (0.8–0.9)	84.5 (77.6–89.9)	75.5 (65.6–83.8)	84.5 (79.1–88.6)	75.5 (67.6–82.1)
dnNMc	0.69 (0.6–0.7)	68.9 (58.7–74.4)	60.8 (50.3–70.6)	72.3 (66.5–77.4)	54.6 (47.7–61.4)
vnNMv	0.83 (0.78–0.88)	85.5 (80.2–90.9)	69.3 (60.3–78.3)	82.1 (76.4–87.8)	74.5 (65.7–83.3)
dnNMv	0.77 (0.71–0.83)	71.7 (64.2–78.4)	68.3 (58.3–77.2)	78.8 (73.3–83.4)	59.5 (52.765.9)
Predefined cutoff vnNMv	0.79 (0.74–0.84)	82.1 (75.5–87.5)	74.5 (64.4–82.9)	85.5 (80.6–89.4)	69.3 (61.6–76.0)

CI, confidence interval; dnNMc, dorsal nigral NM contrast; dnNMv, dorsal nigral NM volume; NM, neuromelanin; SNpc, substantia nigra pars compacta; vnNMc, ventral nigral NM contrast; vnNMv, ventral nigral NM volume.

As expected, dorsal nigral NM metrics showed similar but less pronounced group differences, resulting in lower diagnostic accuracies (Fig. 2; Table 1). Normalized dnNMv and dnNMc were lower in patients, both with adjusted *P* < 0.0001, controlling for sex and age (difference in means [95% CI] of volume and contrast is 62.10 [47.2, 77.00] and 0.24 [0.16, 0.32], respectively). Group differences were also confirmed in post hoc tests in the sex‐matched subsample for dnNMc and dnNMv (see Figure S7 and Table S2 for details).

### Serial Nigral NM Metrics Detect Selective Progressive Depigmentation in PD

**FIG 3:**
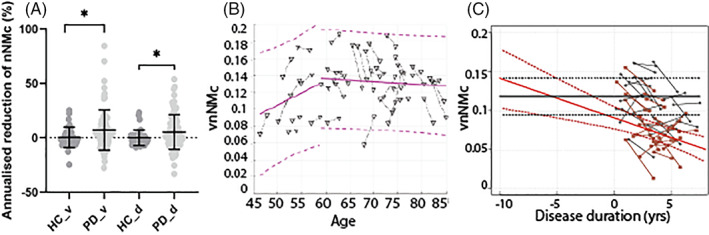
Serial changes in substantia nigra NM‐MRI (neuromelanin‐magnetic resonance imaging) contrast. (**A**) Group‐level decay rates of vnNMc (ventral nigral NM contrast) and dnNMc (dorsal nigral NM contrast) in controls and patients with Parkinson's disease, with individual data presented. Error bars represent mean and SD. * indicates that the difference between groups is statistically significant. (**B**) Scatterplot of individual pairs of serial vnNMc versus age in controls. The black dashed line links nNMc (nigral NM contrast). The pink ascending and descending solid lines represent the best linear fits of the data, respectively, when a predefined age cutoff was applied (pink dashed lines: 95% CI [confidence interval]). (**C**) Linear extrapolation (red solid and dashed lines; mean and 95% CI) of the decay rate in patients, adjusted for sex and age, to the estimated mean of the controls (black solid and dashed lines; mean and 95% CI) suggests about a 5‐ to 6‐year period of ventral nigral depigmentation before clinical (motor) disease onset. The light‐red points and gray points represent baseline–follow‐up time points of vnNMc for patients and controls, respectively.

There was a significant decline in all NM metrics in patients but not in controls. Individual annualized decay rates were significantly higher in PD for vnNMc (patients: −7.3 ± 19.1 vs. controls −0.4 ± 9.3, adjusted *P* = 0.035, Fig. 3A) and dnNMc (patients: −5.3 ± 16.0 vs. controls −0.02 ± 7.1, adjusted *P* = 0.014, Fig. 3A). We also show significantly greater annualized tnNMv loss in PD (−19.2 ± 26.0%) compared to controls (−2.2 ± 28.7%, *P* < 0.05). We did not find a sex difference in the patient groups for annualized decay rates of vnNMc or tnNMv (*P* > 0.05), controlling for disease duration and age.

To further account for a possible sex confound, we repeated the analysis in a sex‐matched subsample. An accelerated NM contrast reduction was confirmed in the sex‐matched sample for vnNMc change rates but not for dorsal SNpc change rates. After removing participants with near‐zero volume at BL, the sex‐ and age‐matched subsample also confirmed accelerated total template‐based nigral NM volume loss in people with PD (see Figure S8 and Table S3 for details).

We then considered possible confounding factors focusing on ventral NM contrast change. In the control group, we assessed age and found a trend increase in vnNMc change with age in those less than a predefined age cutoff (aged 57 years^22^) and with no change or a minor decline in the older subgroup (Fig. 3B). In the patient group, neither age nor disease duration was associated with depigmentation rates (data not shown).

Finally, we extrapolated the decay rate from sex, age, disease duration, and scanner‐adjusted patient metrics to estimate the onset of the premotor depigmentation compared to matched control metrics. The point estimate at group level suggests a 5‐ to 6‐year period of MRI detectable ventral nigral depigmentation before clinical (motor) disease onset (Fig. 3C).

### Nigral NM Metrics and Clinical Findings

**FIG 4:**
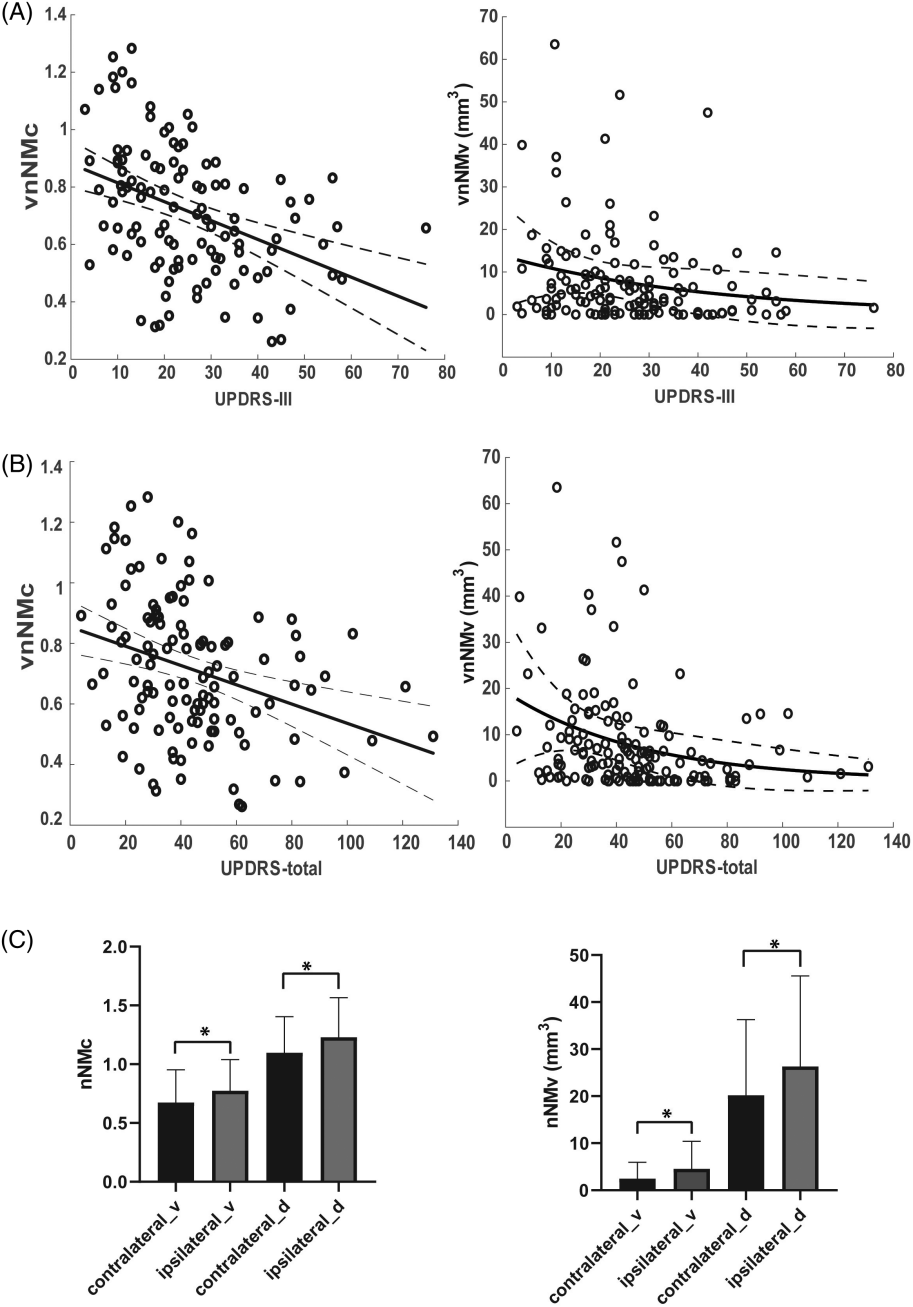
Association between brainstem depigmentation (nNMc and nNMv) and clinical findings: (A) UPDRS‐III (Unified Parkinson's Disease Rating Scale, Part III), (B) UPDRS‐total, and (C) laterality. Solid lines/curves: linear and exponential fittings. Dashed curves: 95% confidence interval {added}. * indicates that the difference between groups is statistically significant. The error bars represent the mean ± standard deviation (SD).

Figure 4A shows anticorrelations of motor severity (UPDRS‐III [Unified Parkinson's Disease Rating Scale, Part III]) with vnNMc (r = −0.36, *P* < 0.0001) and vnNMv (r = −0.20, *P* = 0.02). Similar associations (Fig. 4B) were noted for UPDRS‐total scores (vnNMv: r = −0.29, *P* < 0.0001; vnNMc: r = −0.34, *P* < 0.0001). Both associations of vnNMv (to UPDRS‐III and UPDRS‐total) were better represented by nonlinear curves. No correlations between dnNMc and the clinical information were revealed.

Laterality of NM signal loss was congruent with symptom lateralization. Patients who had an asymmetric affection (n = 75) exhibited reduced NM metrics contralateral versus ipsilateral to the more affected (Fig. 4C).

Serial changes of three NM measures (vnNMc, dnNMc, and tnNMv) were tested against UPDRS‐III change rates. We found that only the reduction rate of tnNMv was positively correlated with the serial increase rate of UPDRS‐III (r = 0.52, *P* < 0.05).

## Discussion

This longitudinal multicenter case–control study in early PD has addressed previously identified limitations of NM‐MRI as a biomarker of nigral depigmentation. Using a published normalization procedure and a dedicated template‐based automated method, we demonstrate a strong disease effect size for ventral nigral NM metrics derived from multisite, multivendor protocols. All tested NM metrics afforded high discriminatory power with good generalizability for a predefined vnNMv threshold. Lower NM metrics were associated with male sex, disease severity, and laterality. The mean annual depigmentation rate in PD, assessed as contrast reduction and total volume loss, was significantly accelerated compared to healthy controls in the full and sex‐matched subsamples. Point estimates at group level support a 5‐ to 6‐year premotor period of in vivo detectable depigmentation in the ventral SN contrast. We also observed large interindividual variability in depigmentation rates, and serial volume changes were associated with progression of motor symptoms. Overall, this study validates automated NM contrast assessment as a powerful tool to quantify and track nigral degeneration in PD in multicenter settings.

Using dedicated 3 T MRI with cross‐site and protocol normalization, we confirmed NM‐related signal loss in the ventral SNpc in early PD, indexed as NM‐rich volume or contrast ratio, and report a high discrimination accuracy of 83%–85%, similar to previously reported accuracy.^14^, ^17^ Importantly, we showed good generalizability of a predefined vnNMv threshold from an independent cohort,^18^ yielding 79% accuracy when applied to the current multicenter data set. The clinical relevance of nigral depigmentation as detected by NM‐MRI was demonstrated by an anticorrelation between disease severity measures and ventral NM metrics and confirmed by a within‐subject laterality effect. In our cohort, only a minority of patients had clinical scores collected *off* medication, which may have masked stronger clinico‐NM associations. Similar associations were previously reported,^14^, ^18^, ^30^ with some inconsistency^15^ that may be explained by technical or population differences.

The small size of SN combining multiple localization and segmentation approaches^11^, ^14^, ^18^ represents a cause of inconsistencies in NM‐MRI findings. Different from previous approaches,^18^ we optimized postprocessing to robustly capture NM‐related signal intensity along the long axis of SNpc. This novel method is automated, which is template‐ and data‐based, making results independent of variations in slice orientation and subjective ROI placement. The resultant oblique line profile highlights an NM gradient with lowest signal in the ventral SNpc. The gradient presented in both groups, but at a lower level with an apparent steeper slope in patients, which is well in line with preferential depigmentation of vnNMc that contains the nigrosome 1, known to be particularly vulnerable to PD.^4^, ^31^, ^32^ These subregional findings also concur with postmortem findings^3^ and a recent voxel‐based NM analysis.^33^

Understanding the effects of demographic factors on NM is important for clinical translation and may pave the way for the assessment of population risk factors. Physiological aging affected NM metrics in this cohort of healthy controls consistent with some previous histochemical reports,^34^, ^35^, ^36^ while the relationship remains controversial (summarized in Gibb and Lees^23^). The lack of an observable age effect on NM metrics in patients is in line with early postmortem data, suggesting that age‐related reduction in pigmented nigral cells is not a major contributor to brainstem depigmentation in PD.^4^ We report a noteworthy sex effect with consistently lower NM metrics in men, in both controls and patients. The underlying mechanism remains unclear, but higher NM content in women may be protective and confer resilience to PD.^37^, ^38^ There is surprisingly little support for sex differences in biomarkers of PD, with one study reporting higher striatal binding on DaTscans in healthy women compared to men^39^, ^40^ and larger volumes in women in a very recent NM‐MRI study.^32^ Other NM‐MRI and DaTscan studies did not detect sex differences^41^, ^42^, ^43^, ^44^ but were generally limited by small sample sizes.

There is an unmet need for a biomarker that can track the progression of PD. The disease trajectory is heterogeneous, and even without the development of disease‐modifying therapies, the ability to track the evolution of disease along individual trajectories would aid in prognostication. It follows that future studies of novel therapies assessed for disease‐modifying effects would benefit enormously from such a biomarker. Therefore, we have quantified annualized depigmentation rates and demonstrate that NM‐MRI can detect disease‐related NM signal loss in PD patients but not in controls, over an interval of 1.5 to 3 years. Annualized change rates varied between individuals and subregions for contrast (5%–7%) but are generally well in line with estimates of the annual loss of striatal dopamine transporter signal in patients (6%–11% per year).^6^

Interestingly, inverse effects with signal increase were reported in a recent longitudinal study in early male PD with a nonsignificant decrease in progressive PD.^32^ In our patient population, mainly including people with less than 5 years disease duration, we did not reveal a sex or disease duration effect on serial NM contrast changes. These differences are most likely due to differences in acquisition and postprocessing relying on more robust and sensitive ventral NM metrics. We also show an accelerated NM volume loss about fivefold higher in PD compared to controls. The volume loss is generally well in line with previous studies reporting volume loss,^30^, ^31^, ^32^ but annualized changes were found to be larger (−19%), which likely reflects the exclusion of participants with advanced depigmentation at BL.

Modeling the depigmentation rate as a function of disease duration, controlled for age, sex, interval, and scanner, we estimate that ventral SN depigmentation precedes the motor diagnosis of PD by about 5 to 6 years. This prediagnostic period of biomarker detectable depigmentation overlaps with estimates for striatal dopamine denervation and possibly earlier than olfactory changes (reviewed in reference^45^). The mixed model assumes a linear decline that fits the data but is in contrast to an exponential decay in dopaminergic innervation based on serial DaTscan,^6^ postmortem estimates of decline in melanized neurons^3^, ^46^ and SN volumetry.^33^ Larger data sets with multiple time points over longer disease spans^47^ are needed for accurate modeling of depigmentation trajectories and their spatiotemporal relationships and associations with disease subgroups.

There are two additional noteworthy factors that may complicate the dynamics of depigmentation markers. First, nigral NM content increases in healthy aging before it declines in older age. In the healthy controls, we show a trend linear vnNMc increase in those younger than 57 years, which is well in line with the previously reported NM signal peak in late adulthood^22^ and highlights the need to correct the complex age–NM relationship in clinical studies as a potential major confounding factor of reported disease‐related NM decline.^25^ Second, the physiological NM–iron complex is not saturated, and the increased free iron in PD may lead to a higher saturation of the NM–iron complex that could initially lead to contrast increase before overt loss of melanized neurons.^12^, ^48^, ^49^, ^50^ These complex interactions may have contributed to the inconsistent results of previous longitudinal MR relaxometric and susceptometric studies^47^, ^51^, ^52^, ^53^ in addition to remaining technical limitations in iron quantification.^54^ By contrast, longitudinal increases in MRI estimates of free water in the ventral SNpc in PD were reported in several cohorts^55^, ^56^; yet underlying mechanisms are to be determined and may lack specificity.^56^, ^57^ On the contrary, the brainstem depigmentation quantified in NM‐MRI provides a highly promising approach to directly monitor the SNpc progressive pathology in PD, which may aid the efficacy assessment of putative disease modifying treatment (DMT).

## Limitations, Outlook, and Strengths

The present study has notable limitations and strengths. There is no histological confirmation as gold standard or comparison with other imaging biomarkers such as DaTscan. Due to the long acquisition times of the multimodal protocols, a substantial amount of data failed quality control due to head motion. Improvement in scan quality could be achieved by shortening the scan protocol, that is, focusing on NM‐MRI, and further use of novel deep‐learning approaches for image reconstruction that can substantially increase signal to noise despite shorter acquisition times. A further limitation is the unbalanced sex between the healthy controls and people with PD. As we reported a clear cross‐sectional effect of sex on NM metrics, we repeated the main analyses in a sex‐matched subsample, which allowed us to confirm the main reported NM disease effects in both cross‐sectional and longitudinal analyses. Also, post hoc tests did not reveal a sex effect on disease‐related serial change of ventral SN contrast. Nevertheless, we cannot fully exclude sex‐related modification of the depigmentation trajectory in PD, which warrants further studies. Although we report high diagnostic accuracy of NM‐MRI, we included participants only with an established clinical diagnosis of PD; therefore, future studies are needed to include subjects at premotor stages and otherwise diagnostically equivocal parkinsonism. The post hoc point estimation of premotor onset of depigmentation of 5 to 6 years is promising to allow the extension, and possibly extrapolation, of preclinical detection and diagnosis of PD in uncertain cases. The current study addressed only clinical phenotypes in the UPDRS scores, but as PD is a multifaceted neurodegeneration disease, other clinical domains, such as cognition and mood, warrant further investigation. There are several strengths of this study, such as its prospective multicenter design with a longitudinal case–control arm and the development and successful application of a standardized template‐based quantification of NM contrast and volume, allowing the robust assessment of subregional and serial nigral depigmentation.

In conclusion, we present multicenter, multiprotocol, and multivendor quantitative MRI evidence of nigral depigmentation and a significant sex effect in PD with clinical useful diagnostic accuracy and report accelerated annualized depigmentation rates using a fully automated quantification approach.

## Author Roles

Study concept and design: all authors

Acquisition, analysis, or interpretation of data: all authors

Drafting of the manuscript: all authors

Editing the manuscript: Y.X. and D.P.A.

Critical revision of the manuscript for important intellectual content: all authors

Statistical analysis: Y.X., C.T., and D.P.A.

Administrative, technical, or material support: all authors

Study supervision: D.P.A.

## Financial disclosures for the past 12 months

Relevant conflicts of interest/financial disclosures: Y.X. and D.P.A. received support from Biogen.

## Supporting information


**Appendix S1**. Supporting Information

## Data Availability

The data that support the findings of this study are available from the corresponding author upon reasonable request.
