# Supplementary material for: Neuromelanin‐MRI to Quantify and Track Nigral Depigmentation in Parkinson's Disease: A Multicenter Longitudinal Study Using Template‐Based Standardized Analysis
Source: Mov Disord. 2022 Feb 15;37(5):1028–39. doi: 10.1002/mds.28934 (PMC9303322; doi:10.1002/mds.28934)
Supplement: Supplementary file 1 — Appendix S1. Supporting Information [file MDS-37-1028-s001.docx]

**Supplementary material:**

***1.1 Tracking Parkinson’s***

The ProBaND study, ‘PRoBaND: Parkinson’s Repository of Biosamples and Network Datasets’, MREC No: 11/AL/0163, UKCRN ID: 11769, Sponsors: NHS Greater Glasgow & Clyde and The University of Glasgow.

***1.2 Pathways for recruiting healthy controls***

**
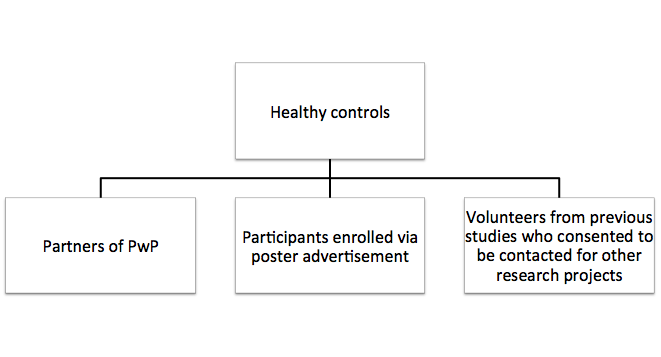
**

***1.3 The inclusion and exclusion criteria of ProBaND study in addition to the PaMIR criteria related to the MRI scanning***

1. Parkinson’s disease patients:

Inclusion criteria

1. Diagnosis of Parkinson's disease, based on UK Brain Bank criteria
2. Age ≥18 to <90years
3. Subject is able and willing to provided informed consent.

Exclusion criteria

1. Patient has severe comorbid illness that would prevent full study participation
2. Patient has features indicating another type of degenerative parkinsonism, e.g. progressive supranuclear palsy
3. Drug-induced parkinsonism (Drug-unmasked PD is allowed)
4. Symmetrical lower body parkinsonism attributable to significant cortical and/or subcortical cerebrovascular disease (patients with 'incidental' small vessel disease on brain imaging are allowed).
5. Negative or normal functional imaging of the presynaptic dopamine system
6. The presence of UK Brain Bank exclusion criteria will be recorded at baseline, allowing for the presence of 1 or 2 exclusion criteria (e.g. dopamine antagonist Drug used; more than one affected relative) (if justified e.g. by abnormal SPECT).
7. Any contraindication to Magnetic Resonance (MR) scanning.
8. Any major neurological (other than PD), psychiatric or cardiovascular disease or history of brain injury.
9. Medical illness or medication that may affect brain morphometry of function.
10. For Healthy controls

Inclusion criteria

1. Age ≥18 to < 90years
2. Subject is able and willing to provided informed consent.

Exclusion criteria

1. Subject has severe comorbid illness that would prevent study participation
2. Subject already has a diagnosis of Parkinson's disease

***1.4 Summary of the PaMIR core MRI protocol and the NM-MRI sequences used for different sites***

**1.4.1** In addition to NM-MRI, the PaMIR study also includes MRI sequences as follows: T1-weighted imaging, susceptibility weighted imaging, resting-state functional MRI, and diffusion tensor imaging.

**1.4.2** All the NM-MRI was T1-weighted imaging and S. Table 1 lists the NM-MRI sequences related information at all sites

S. Table 1 Information of the scanner and the MRI sequences in different sites

| **Site** | **Participants** | **Scanner platform and head coil channels** | **Neuromelanin-MRI Sequence Parameters** | **T1 structural MRI Sequence Parameters** |
| --- | --- | --- | --- | --- |
| Nottingham | 19 controls and 26 patients with PD | 3T GE scanner (Discovery MR750; software version: DV24; 32-channel | Nottingham sequence-1:  T1-weighted spin-echo sequence with additional “off-resonance” MT pulse: TR, 600 ms; TE: 10 ms; slice thickness, 2.5 mm; in-plane resolution, 0.38mm*0.38mm; slice gap, 0.3mm; three averages; slices number, 12/11; scanning time, 9.32 minutes | A whole-brain high-resolution axial T1-weighted 3D FSPGR scan: TR, 8.5 ms; TE, 3.3 ms; inversion time, 450 ms; Acceleration factor = 2; voxel size, 1 isotropic mm^3^. |
|  | 37 controls and 35 patients with PD | 3T GE scanner (Discovery MR750; GE Healthcare, Milwaukee, WI); 32-channel | Nottingham sequence-2:  3D‐dimensional spoiled gradient recalled T1‐weighted sequence:  Repetition time (TR), 38.4 milliseconds (ms); Echo time (TE), 3 ms; slice thickness, 2mm; in-plane resolution, 0.375mm*0.375mm; no gap; slices number, 30; scanning time, 3.25 minutes |  |
| London | 10 controls and 21 patients with PD | 3T Siemens Magnetom, TrioTim MR, 32-channel | T_1_-weighted turbo spin echo sequence: TR, 829 ms; TE, 12ms; low specific absorption rate; slice thickness, 2.5mm; in-plane resolution, 0.4mm*0.4mm; no gap; Echo train length=4; echo spacing: 11.5 ms; slices number, 12; scanning time, 5.24 minutes | A whole-brain high resolution volumetric T_1_-weighted magnetization prepared rapid acquisition gradient echo (MPRAGE): TR, 2300 ms; TE, 2.98 ms; time to inversion = 900 ms; GRAPPA acceleration factor PE = 2; voxel size, 1 isotropic mm^3^. |
| Newcastle | 6 controls and 20 patients with PD | 3T Philips  Achieva  Software versions: 5.1.7_5.1.7.1; 8-channel | T1-weighted sequence with a standard Philips “off-resonance” MT pulse: TR, 1050 ms; TE: 9ms, in-plane resolution, 0.8mm*0.8mm; slice thickness, 2.5 mm; slice gap: 0.3mm; Echo train length=3; slices number, 13; scanning time, 6.49 minutes | A whole-brain high resolution 3D T1- weighted spin echo SENSE sequence:  TR, 25 ms; TE, 1.7 ms; voxel size, 1 isotropic mm^3^. |
| Manchester | 13 controls and 20 patients with PD | 3T Philips  Achieva; 8-channel | T1-weighted sequence with a standard Philips “off-resonance” MT pulse: TR, 1050 ms; TE: 9ms, in-plane resolution, 0.8mm*0.8mm; slice thickness, 2.5 mm; slice gap: 0.3mm; Echo train length=3; slices number, 13; scanning time, 6.49 minutes | A whole-brain high resolution 3D T1- weighted spin echo SENSE sequence:  TR, 25 ms; TE, 1.7 ms; voxel size, 1 isotropic mm^3^. |
| Glasgow | 8 Controls and 12 patients with PD | 3T GE scanner Signa_HDxt 32-channel | Glasgow sequence-1:  T1-weighted spin-echo sequence with additional “off-resonance” MT pulse: TR, 600 ms; TE: 10 ms; slice thickness, 2.5 mm; in-plane resolution, 0.38mm*0.38mm; slice gap, 0.3mm; three averages; slices number, 12/11; scanning time, 9.32 minutes | A whole-brain high-resolution axial T1-weighted 3D FSPGR sequence: TR, 8.5 ms; TE, 3.3 ms; inversion time, 450 ms; Acceleration factor = 2; voxel size, 1 isotropic mm^3^. |
|  | 4 Controls and 14 patients with PD | 3T Siemens Prisma,  Syngo MR; 32 Channel | Glasgow sequence-2:  T_1_-weighted turbo spin echo sequence: TR, 829 ms; TE, 12ms; low specific absorption rate; slice thickness, 2.5mm; in-plane resolution, 0.4mm*0.4mm; no gap; Echo train length=4; echo spacing: 11.5 ms; slices number, 12; scanning time, 5.24 minutes | A whole-brain high resolution volumetric T_1_-weighted magnetization prepared rapid acquisition gradient echo (MPRAGE): TR, 2300 ms; TE, 2.98 ms; time to inversion = 900 ms; GRAPPA acceleration factor PE = 2; voxel size, 1 isotropic mm^3^. |


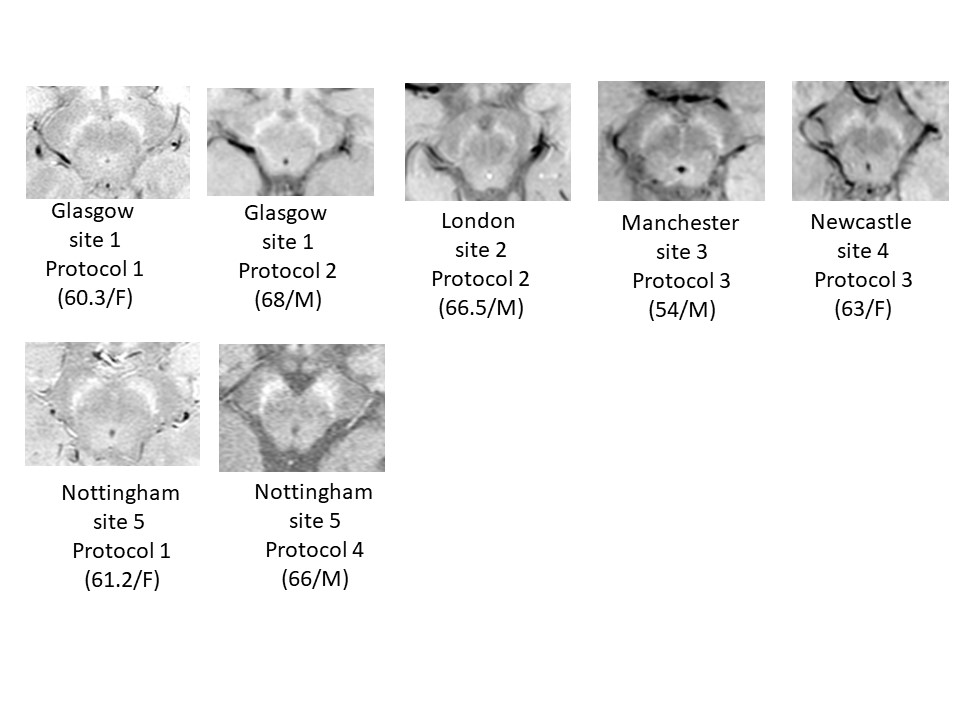


S. Figure 1: Example images from each site and protocols

***1.5 Template-based NM SN contrast assessment***

First, the structural images of 27 HC subjects from different sites were selected and affinely registered to a standard brainstem space (ICBM152 space, http://www.bic.mni.mcgill.ca/ServicesAtlases/ICBM152NLin2009). Their NM-MRIs were linearly registered to the individual T1-weighted image, which were then transformed to the standard space. This generates an age specific healthy control’s mean brainstem template, along with their respective spatial priori probability maps with three classes: brainstem outside of the SN, SN and irrelevant background. The same procedure was performed to transform NM-MRIs in the same space as the prior probability maps. Finally, the Bayesian classification was applied for partitioning the SN from the brainstem in both image intensity and in space and the mean intensity of the brainstem was extracted.


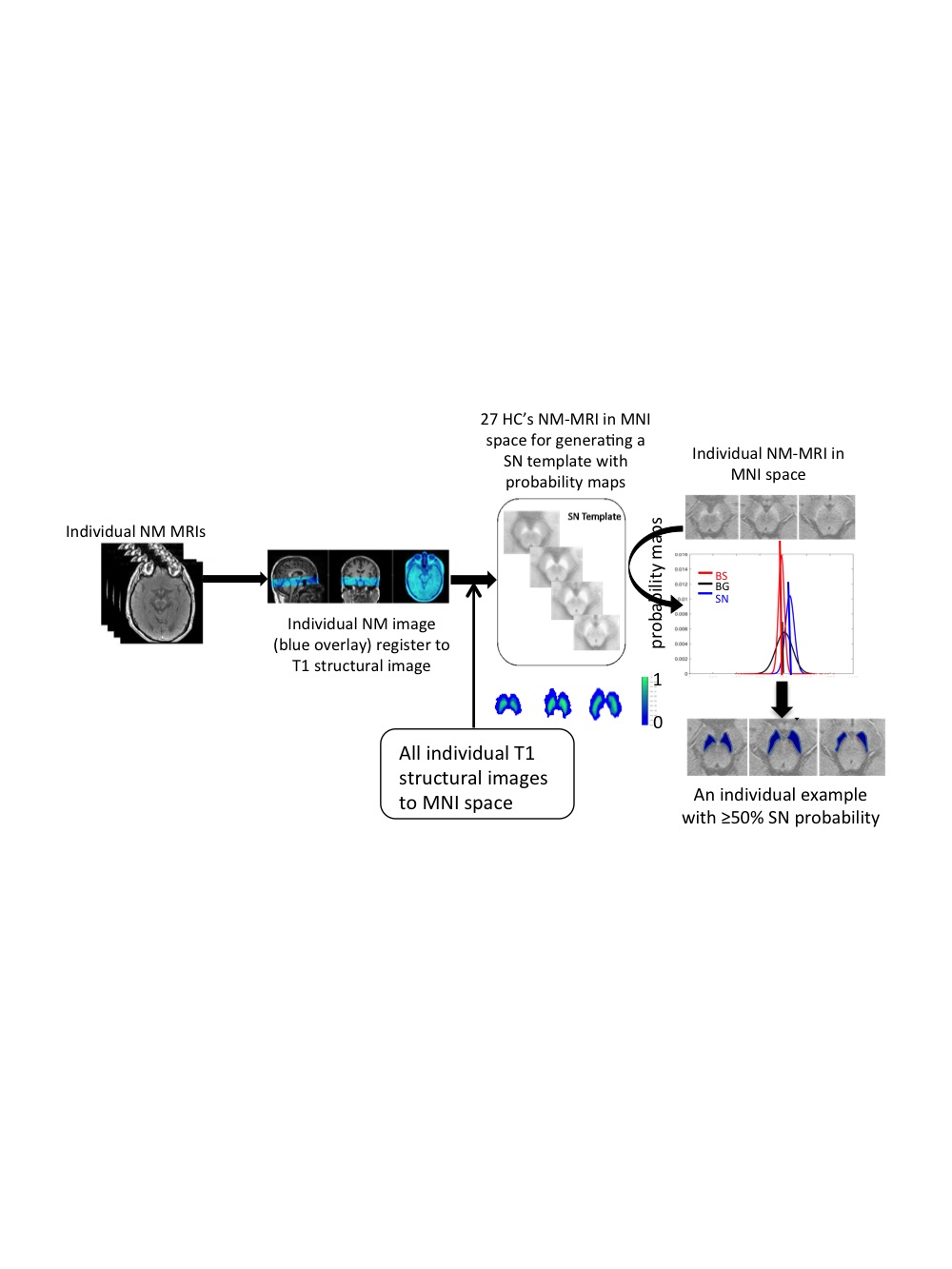


S. Figure 2. Illustration of the analysis pipeline: NM-MRIs were linearly registered to the individual T1, which were then transformed to the same space as the healthy SN template. Based on the prior probability of the SN (blue probability map of 3 slices, ranging from 0 to 1), the brainstem area outside SN (BS), and the irrelevant background outside brainstem (BG), we applied the Bayesian approach and obtained the respective spatial priori probability of these three classes of tissue for each individual. The highlighted blue areas in the last graph of the pipeline are areas above 50% posterior probability map of SN.


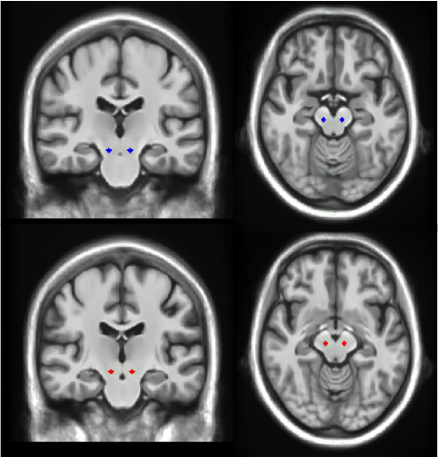


S. Figure 3. The positions of regions of interest in the substantia nigra based on the ICBM152 template space: bilateral dorsal SNpc: right (107 115 60) and left (89 115 60); ventral SNpc: right (107 112 57) and left dSNpc (89 112 57).


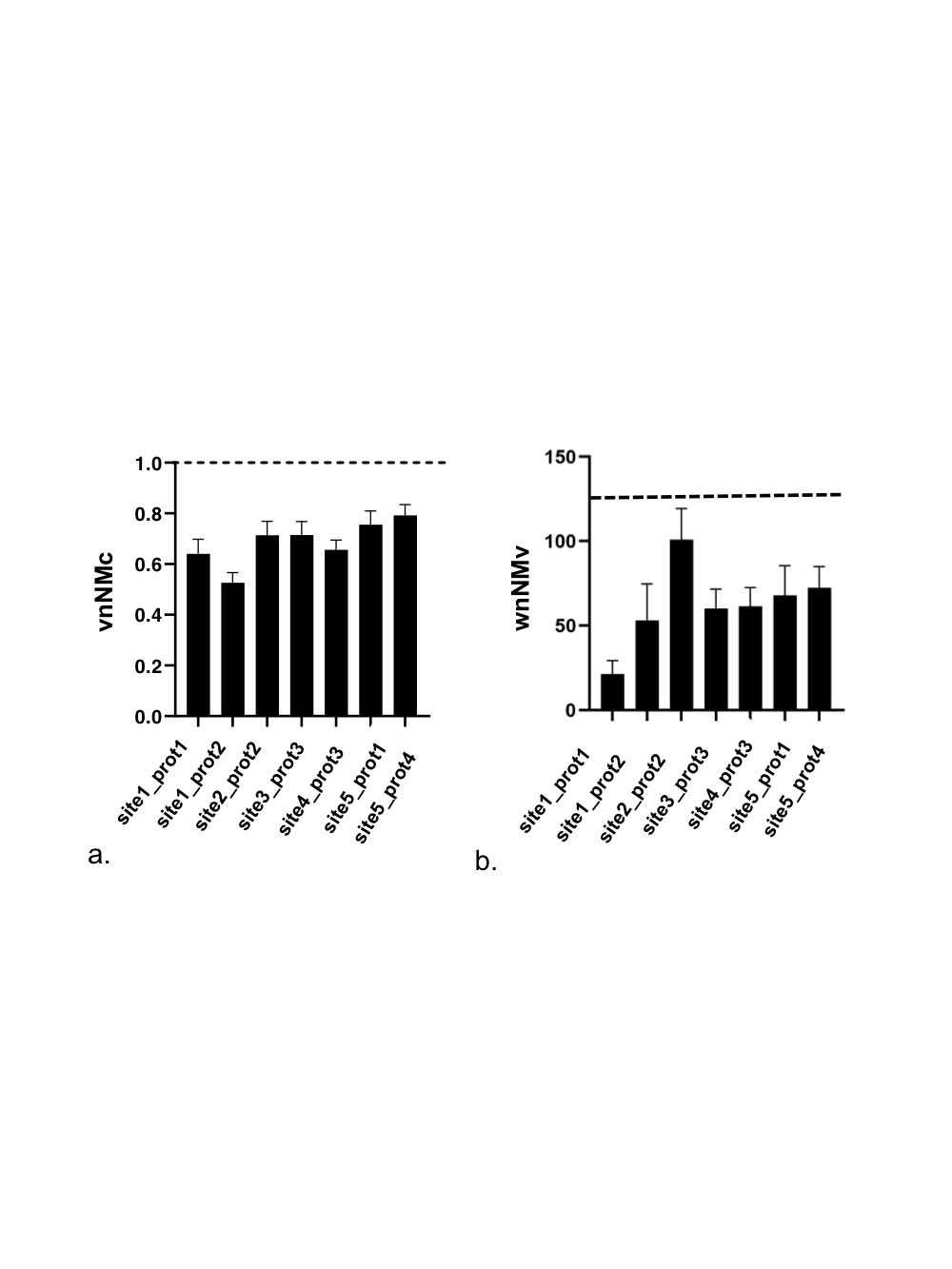


S. Figure 4. The variation of normalised vnNMc (a) and wnNMv (b) between protocols and sites in PD (dash line in a. represents the normalised HC value=1; and dash line in b. represents the normalised HC value=127mm^3^). 4 protocols and 5 sites were involved in the study.

***1.6 Statistical methods used for testing the relation of nigral neuromelanin metrics to clinical findings***

For the fitting of NM-clinical measures, we used linearly or nonlinear fitting function polyfit in Matlab. Moreover, we studied the laterality of NM changes in Parkinson’s brains. To determine the predominately-affected side of limbs in patients, an index of the right minus left scores from all the MDS-UPDRS III items associated with the motor lateralization was calculated. A 3-points difference was used as the threshold, and if right-left>3, then right is the predominately affected side and vice versa (Uitti et al., 2005; Barrett et al., 2011). To avoid the possible effect of handedness, we only chose the patients with right-handedness, which was determined using the Edinburgh Handedness Inventory (Oldfield, 1971). Contralateral vs. ipsilateral nigral NM contrast and volumetry were compared using paired t-test. The asymmetry of NM in right-handedness HC was also examined by comparing left and right hemispheric nNMv and nNMc (dorsal and ventral tiers), respectively using the same statistical method.

***1.7 Image quality control and exclusion criteria***

Quality control for NM: All the imaging slices that contained the SN were visually inspected by two viewers blinded to clinical and demographic information. NM images considered to be degraded by body motion artefacts, motion/vibration artefact from the surrounding vessels and/or significant local signal drop-off were removed from further analysis after reaching consensus between the two raters. Examples were illustrated in Figures below.


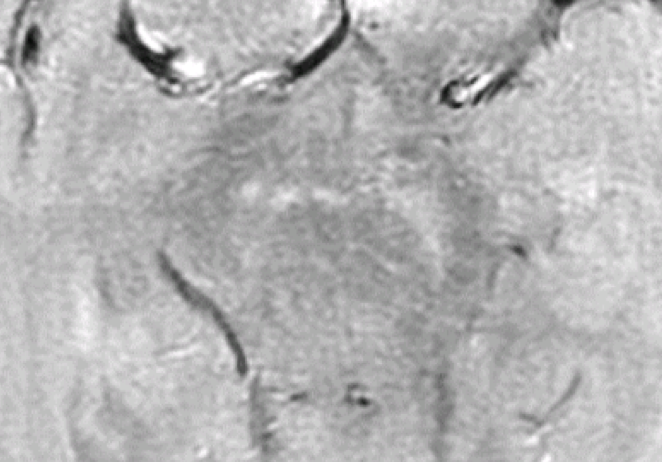

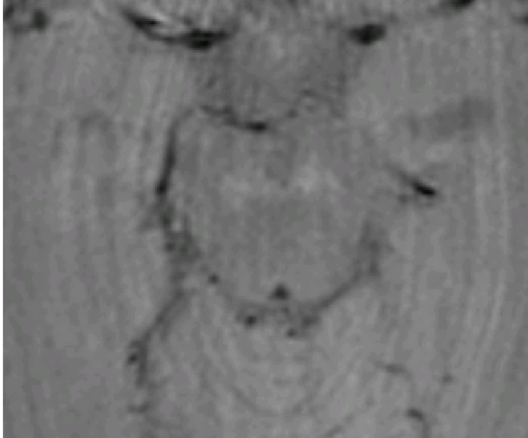


S. Figure 5: The left NM-MRI showed serious head movement, which affected the extraction of normal signal intensity of the background regions. The right image presented high intensity pulsation artefacts (yellow arrows), which confuse the delineation of SN, particularly the right SN.


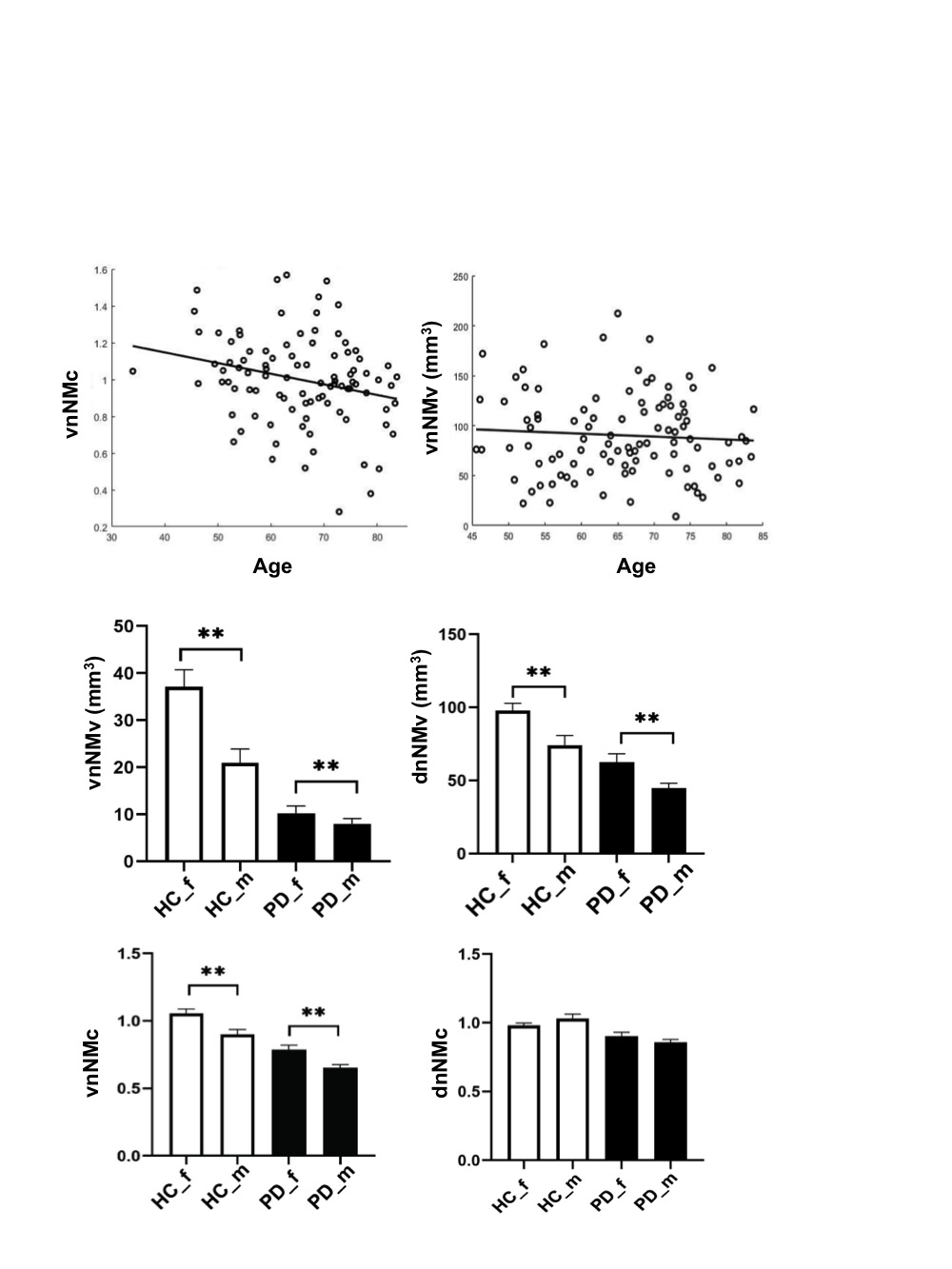


S. Figure 6: Age effect (upper panel) and sex difference of volumetry (middle panel) and nigral NM contrast (bottom panel). *Upper panel*: the scattered plots and fitted straight lines showed negative correlations between normal aging and NM metrics, suggesting that both vnNMc (left) and vnNMv (right) decreased as age increased in HC. *Middle panel*: Female subjects (f) demonstrated larger NM volumes in both ventral (left) and dorsal (right) SNpc than male subjects (m) in controls and PD. *Bottom panel:* Female subjects (f) showed higher nNMc than male subjects (m) in both controls and PD in ventral part (left) of SN but not dorsal part (right). Error bars: mean±SEM, **significant group difference
